# Transcription factor interplay in T helper cell differentiation

**DOI:** 10.1093/bfgp/elt025

**Published:** 2013-07-22

**Authors:** Catherine M. Evans, Richard G. Jenner

**Keywords:** T cell, transcription factor, lineage-specification, cell differentiation, plasticity, enhancer

## Abstract

The differentiation of CD4 helper T cells into specialized effector lineages has provided a powerful model for understanding immune cell differentiation. Distinct lineages have been defined by differential expression of signature cytokines and the lineage-specifying transcription factors necessary and sufficient for their production. The traditional paradigm of differentiation towards Th1 and Th2 subtypes driven by T-bet and GATA3, respectively, has been extended to incorporate additional T cell lineages and transcriptional regulators. Technological advances have expanded our view of these lineage-specifying transcription factors to the whole genome and revealed unexpected interplay between them. From these data, it is becoming clear that lineage specification is more complex and plastic than previous models might have suggested. Here, we present an overview of the different forms of transcription factor interplay that have been identified and how T cell phenotypes arise as a product of this interplay within complex regulatory networks. We also suggest experimental strategies that will provide further insight into the mechanisms that underlie T cell lineage specification and plasticity.

## INTRODUCTION

### T helper cell differentiation and lineages: The Th1/Th2 paradigm

Differentiation of naïve CD4+ T cells into different helper subtypes allows tailoring of the immune response to different pathogens and constitutes a powerful model system for the study of cell specification. Different helper T cells function in part through the secretion of cytokines that modulate the function of other immune cell types. Differential production of these cytokines has provided the foundation for helper T cell classification from which transcriptional regulatory mechanisms have been characterized. The definition of different helper T cell subtypes began with the description of two distinct classes that exhibited differences in cytokine production that were stable during passage [1]. This demonstrated that the specialization of helper T cells was deterministic rather than stochastic and led to the Th1–Th2 paradigm for differentiation of specialized T helper lineages. Under this paradigm, Th1 cells, which differentiate in the presence of IFNγ and IL-12, produce the signature cytokine IFNγ to activate macrophages and cytotoxic CD8+ cells to clear intracellular pathogens. Th2 cell differentiation is induced by IL-4 and the cells also secrete this cytokine, leading to activation of the humoral immune response and clearance of extracellular parasites [2]. The concept that Th1 and Th2 cells are separate lineages was reinforced by the discovery of the transcriptional ‘master regulators’ T-bet (TBX21) [3] and GATA3 [4] as being necessary and sufficient for the development of Th1 and Th2 cells, respectively. T-bet was found to be specifically induced in Th1 cells, in which it binds and activates *Ifng*, whereas GATA3 in Th2 cells binds and activates the *Il4/Il5/Il13* cytokine locus. Other T-box and GATA factors play key roles in embryonic development, such as the heart and limbs, suggesting parallels between classical developmental processes and T cell activation in adult mammals [5].

### Growing numbers of helper T cell subtypes and lineage plasticity

The Th1–Th2 paradigm was subsequently extended to encompass a number of additional subsets. Th17 cells, named for their signature production of IL-17, differentiate through TGF-β and IL-6 signalling and clear extracellular bacteria and fungi [2]. In contrast, induced regulatory T cells (iTreg) differentiate under TGF-β and IL-2 signalling and suppress immune responses [6]. An additional subset of CD4+ Treg, naturally occurring Treg (nTreg), exit the thymus in parallel to naïve CD4 T helper precursors [7]. Each of these subsets is associated with an immune pathology when their differentiation and function is dysregulated [8] suggesting they play key individual roles in the immune response. ‘Master regulator’ transcription factors have also been identified for these lineages; RORγt for Th17 [9], FOXP3 for iTreg and nTreg [10–12]. Follicular helper T (Tfh) cells, which express the ‘master regulator’ BCL6 [13–15], Th9 [16] and Th22 [17] cell lineages have also been described.

Recent findings also suggest that these T cell lineages may not be as immutable as once thought. For example, Th17 cells can become exclusive IFNγ producers and Tfh cells can be re-differentiated to make IFNγ, IL-4 or IL-17 (reviewed in [18–20]). Tregs can also convert to effector (non-regulatory helper) cells in inflammatory environments. Even stable GATA3 expressing Th2 cells can acquire Th1 functionality after transfer to mice subsequently infected with LCMV [21]. Such plasticity may reflect the frequent co-expression of what had been considered lineage-specific master regulatory transcription factors. For example, FOXP3 has been found to be co-expressed with GATA3, T-bet or RORγt and GATA3 and T-bet have been found together within the same cells (Table 1).

**Table 1:** Co-expressed lineage-specifying transcription factors

Factor 1	Factor 2	Cell phenotype	Condition	References
FOXP3	GATA3	Treg	*In vivo* during steady state.	[22]
		Treg	GI tract and skin during inflammation. Human *in vitro* following TCR engagement.	[23]
		Treg	Upon TCR stimulation with IL-*2 in vitro.*	[24]
FOXP3	T-bet	Treg (Th1–Treg intermediate)	*In vivo* during Th1-polarizing infection.	[25–27]
		Treg	In response to IFNγ and IL-27 during Th1-polarizing infection.	[28]
FOXP3	RORγt	Th17–Treg intermediate	Lamina propria and *in vitro* in response to TGF-β and TCR stimulation.	[29, 30]
		Treg	*Ex vivo* during steady state, intestinal inflammation, viral infection and cancer.	[31]
		Treg–Th17 intermediate	*In vivo* in autoimmune diabetes model (NOD).	[32]
FOXP3	BCL6	Follicular Treg	In germinal centres after immunization with antigen.	[33, 34]
T-bet	BCL6	Tfh–Th1 transitional state	In germinal centres during Th1-polarizing infection.	[35, 36]
		Th1	During *in vitro* Th1 differentiation (when IL-2 is limiting).	[37, 38]
T-bet	GATA3	Th1	During *in vitro* differentiation of human cells.	[39–41,]
		Th1	Human *ex vivo* steady state and in *in vitro* generated Th2 clones upon TCR stimulation.	[42]
		Th2 + 1	*In vivo* after transfer of Th2 cells to mice subsequently infected with LCMV and *in vitro* through IL-12 and type I and II IFN signalling.	[21]
T-bet	RORγt	Th17	Human *in vitro* upon TCR stimulation.	[43]
		Th17	*In vitro* with IL-23, IL-6 and IL-1β and *in vivo* in a brain inflammation model (EAE).	[44]
		Th17/1 intermediate	Cells from autoimmune juvenile inflammatory arthritis patients *ex vivo*.	[45]
		Th17	Human *in vitro* after priming with *Candida albicans*.	[46]
		Th17	*In vitro* with TGF-β3 and IL-6 signalling.	[47]
GATA3	RORγt	Th2 memory	Cells from allergic asthma patients *ex vivo*.	[48]

All are mouse unless otherwise specified. GI tract, gastrointestinal tract; MS, multiple sclerosis; EAE; experimental allergic encephalomyelitis; NOD, non-obese diabetic; LCMV, lymphocytic choriomeningitis virus.

The continued identification of T cell subsets and the plasticity that exists between them has led to debate over the true meaning of a T cell lineage. When viewed from a transcriptional regulation stand-point, it would appear that the assumption that expression of a transcription factor equates to a specific phenotype can be misleading. The concept of ‘master regulator’ transcription factors, although useful in identifying critical regulatory factors would seem to underestimate the complexity of helper T cell function. To understand how cell phenotype arises, we must first understand the interplay between the multiple transcription factors that are co-expressed within the cell.

Functional genomics approaches are playing a key role in this work. No longer limited to the study of a small number of cytokine and cell surface markers, T cell phenotypes can now be characterized and understood through measurements of gene expression, epigenetic modifications and chromatin structure at a genomic level. This has expanded our understanding of transcription factor function from the signature cytokines to the rest of the genome and illustrated how T cell differentiation truly is a genomic event.

**Figure 1:**
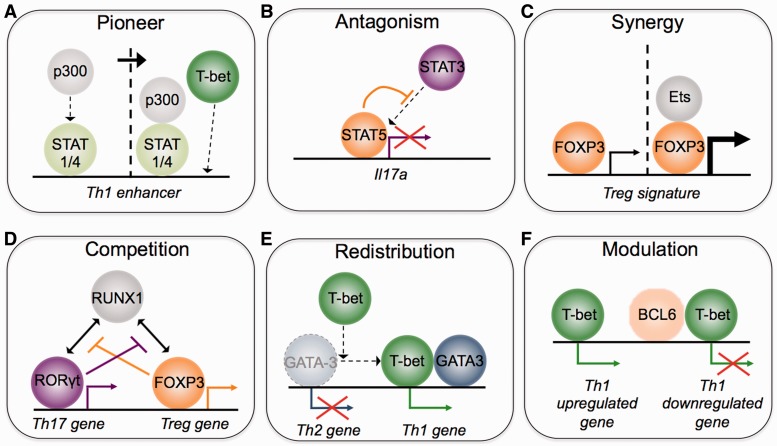
Modes of transcription factor interplay. (**A**) Pioneering transcription factors prepare the epigenetic landscape, allowing other factors to bind to regulatory elements. For example, STAT1 and STAT4 allow the subsequent binding of T-bet and Th1 differentiation. (**B**) Transcription factors can antagonize the function of others, for example, STAT5, associated with Treg differentiation, suppresses Th17 cell function by blocking STAT3 binding and activation of the *Il17a* locus. (**C**) Lineage-specific transcription factors, such as FOXP3, synergize with co-factors (such as Ets) to enhance gene expression and produce a more robust T cell subset signature. (**D**) Competition for a mutual co-factor. For example RORγt and FOXP3 compete for binding to RUNX1, inhibiting each other’s activity. (**E**) Redistribution of a factor to new sites. T-bet sequesters GATA3 away from its Th2 target genes and redistributes it to Th1-associated T-bet targets. (**F**) The activity of a transcription factor can be modulated by other factors, for example, repressive activity is endowed upon T-bet by BCL6.

A number of modes of transcription factor interplay are evident from this data (Figure 1); factors binding early and acting as pioneers for the lineage-specifying proteins (Figure 1A), antagonism expressed as mutually exclusive binding to the same site (Figure 1B), synergism between factors (Figure 1C), competition for a shared co-factor (Figure 1D), redistribution of one factor by another (Figure 1E) and modulation of a regulators activity by another (Figure 1F).

## PIONEER FACTORS

Different cell types arise in development from the differential use of regulatory elements such as enhancers and it would seem reasonable to assume that the lineage-specifying factors such as T-bet, GATA3 and FOXP3 initiate this cell-type specific enhancer activation. But instead it appears that other pioneering factors function to first set up these enhancer repertoires (Figure 1A).

The induction of lineage-specifying transcription factors is initiated by cytokines that signal through receptors to activate signal transducer and activator and transcription (STAT) family members, thereby directly linking the cytokine environment to transcriptional regulation. Different cytokines lead to activation of different STATs, which begin the specification of different lineages. As a simplistic model, STAT4 (activated by IL-12 [49]) leads to Th1 differentiation (STAT1, activated by IFNγ, also plays a role [50]), STAT6 (activated by IL-4 [51]) to Th2, STAT3 (activated by IL-6, IL-21 and IL-23) to Th17 [52], and STAT5 (activated by IL-2) to Treg [53]. STATs bind at genes encoding lineage-specifying cytokines and transcription factors and are necessary for their expression and can thereby be considered as initiating the process of lineage specification. Functional genomics studies have revealed that STATs are also vital for determining enhancer function across the genome [54–56]. Using ChIP-Seq, Vahedi *et al.* [55] revealed that Th1 enhancer elements (defined by Th1-specific p300 binding in the absence of H3K4me3) were enriched for binding of STAT1 and STAT4 in Th1 cells and instead for STAT6 in Th2 cells. Consistent with a role for STATs in generating these enhancer landscapes, p300 binding was dependent on STAT1 or STAT4 at over half of Th1 enhancers, and on STAT6 at three-quarters of Th2 enhancers [55]. Similarly, STAT3 is required for p300 binding at regulatory elements in Th17 cells [56].

STATs appear to play a more fundamental role in lineage-specifying enhancer activity than the lineage-specifying factors that are subsequently upregulated. P300 binding was only found to be dependent on T-bet at 17% of Th1-specific enhancers, compared to 58% being dependent on either STAT1 or STAT4. Furthermore, overexpression of T-bet only recovered p300 binding at 23% of Th1-specific STAT4-dependent enhancers [55]. Similarly, RORγt has a limited effect on p300 binding and on H3K4 di- and tri-methylation [56].

However, arguing against an all-encompassing role for STATs in defining enhancer activity, H3K4me1 at these sites is not dependent on STATs [55] and only one-quarter of STAT-bound genes exhibit STAT4- or STAT6-dependent histone H3 lysine 4 trimethylation (H3K4me3; initiation), K27me3 (poised repression) or K36me3 (elongation) [54], suggesting they are not sufficient for normal patterns of epigenetic modification and gene activity. Other factors, such as AP-1 and NFAT family members, which are activated downstream of the T cell receptor (TCR), have been implicated in functioning as general acting pioneer factors at enhancers in Th1 and Th2 cells [55], Th17 cells [56] and in Tregs [57]. In addition, IRF4 and BATF are important for initiating enhancer activity (measured by p300 binding and open chromatin) in non-polarized cells, at which STAT3 and RORγt then bind in Th17 cells [56]. Indeed, RORγt was found to be almost exclusively bound to sites also bound by IRF4, BATF and STAT3 [56].

Transcription factors of the same structural family can also act as pioneer factors for the lineage-specifying factors. Samstein *et al.* [57] found that 98% of FOXP3 binding sites were already accessible (DNaseI hypersensitive) in FOXP3-negative CD4 cells. However, this doesn’t rule out that FOXP3 has other effects on enhancer function subsequent to formation of an open chromatin structure. Noting enrichment of Forkhead motifs at these sites and evidence that another Forkhead family member, FOXO1, also functions in Tregs, the authors found that FOXO1 acts as a placeholder for FOXP3, occupying sites in FOXP3-negative CD4 T cells that are subsequently bound by FOXP3. Moreover, FOXP3 binding resulted in reduced FOXO1 binding and a reduction in gene expression, suggesting that FOXP3 displaces the FOXO1 placeholder. Samstein and colleagues also identified binding of ELF1, ETS1 and Runx/Cbfβ at FOXP3 pre-accessible sites. These proteins interact with FOXP3 [24], suggesting that co-factors can also act as placeholders for the subsequent binding of lineage-specifying factors.

Taken together, these studies suggest that the major epigenetic changes that occur during T cell polarization take place downstream of TCR ligation and cytokine signalling before the lineage-specifying factors are induced. The lineage-specifying factors would then seem to act within this already established epigenetic landscape to drive a smaller set of more focused changes in gene expression pertinent to the specific role of that particular lineage. This is consistent with gene expression profiling data which shows that the greatest extent of transcriptome remodelling occurs during the early phases of T cell activation [58, 59] and provides a potential molecular basis for lineage plasticity.

### Interplay between pioneer factors

TCR signalling occurs in concert with signals from other receptors and, *in vivo,* cytokines are not provided to cells in isolation as they are during *in vitro* experimental lineage specification. It is, therefore, important to understand the interplay between pioneer factors activated by these different pathways.

Comparison between ChIP-Seq datasets shows that pioneer factors tend to bind to the same genes, often at the same sites. As described above, FOXO1, ETS1, ELF1 and Runx/Cbfβ are often found at the same positions along the genome in Treg precursors [57] and IRF4, BATF and subsequently STAT3 bind to the same sites in Th17 cells [56]. Although functioning in different differentiation pathways, ∼50% of STAT4 (Th1) and STAT6 (Th2) target genes are bound by the other factor [54].

This shared binding can be both cooperative and antagonistic. Both STAT4 and STAT1 induce T-bet expression [50, 60]. Similarly, STAT5A/5B bind to Th2 genes in addition to STAT6 [61–63] and collaborate with STAT6 for the induction of Il4 production and Th2-mediated inflammation [64]. Indeed, two-thirds of genes directly regulated by STAT6 were found to also be regulated by STAT5A [65]. Similarly, in Th17 cells, ChIP in cells from genetically deficient mice reveals BATF and IRF4 binding to be co-dependent [56].

In the context of Th17 differentiation, STAT5 has an antagonistic relationship with STAT3 [66]. STAT5 (induced by IL-2) competes with STAT3 (induced by IL-6) for binding to *Il17a*, leading to loss of permissive histone modifications and a reduction in expression (Figure 1B—Antagonism). The resultant expression level of *Il17a* depends on the relative concentrations of IL-2 and IL-6. However, in the absence of TGF-β and in the presence of IL-4, STAT3 acts cooperatively, being required for STAT6 binding to sites at Th2 genes [67]. In this way, the interplay between the different STATs allows the cell to sense complexities in the cytokine environment and the resultant cell phenotype could lie somewhere along a continuum rather than representing a decisive differentiation decision.

## LINEAGE-SPECIFYING REGULATORS OPERATE WITH CO-FACTORS AND IN NETWORKS

Once the chromatin landscape has been prepared by the pioneer transcription factors, the lineage-specifying transcription factors take effect. These proteins are associated with both the activation and repression of target genes and these targets can vary between cells. These differences in transcription factor activity in different contexts are partly related to the presence of co-factors, which can synergize with or modulate lineage-specifying factor activity. Co-factors that functionally cooperate with each of the lineage-specific factors have been identified. Although in some cases evidence of physical interaction or complex formation is lacking, these co-factors are necessary for optimal activation or repression of lineage-specific target genes. These co-factors include ETS1, HLX and RUNX3 for T-bet [68–70], GFI1, cMAF and DEC2 for GATA3 [71–74] and RUNX1, BATF, IRF4 and FOSL1 for RORγt [56, 75, 76].

### Transcriptional regulatory networks in Tregs

Although the phenotypes caused by FOXP3-deficiency suggest it is the ‘master regulator’ for Treg cells, its interaction with co-factors seems to be especially important for generation of the full Treg gene expression program (Figure 1C—Synergy). Rudra and colleagues [24] used biochemical methods to identify the FOXP3 interactome. Over 300 FOXP3 potential co-factors were identified by mass spectrometry. Of these, 27% had known roles in transcriptional regulation. These included transcription factors, such as RUNX1, NFATc2, FOXP1, GATA3, STAT3, Ikaros, Aiolos and Ets, many of which have previously been implicated in Treg differentiation and co-occupy sites with FOXP3 [57, 24]. Rudra *et al.* also noted that members of this FOXP3 interactome target the *Foxp3* gene and, reciprocally, that FOXP3 targets the genes encoding its partner proteins.

Similar results were gathered by Fu and co-workers, who used the context likelihood of relatedness algorithm to ‘reverse-engineer’ the Treg transcriptional network and to identify the relationship between FOXP3 and other transcription factors [77]. By comparing the gene expression profiles of Treg and other helper T cells from various anatomical locations and varying phenotypes, the authors identified a set of transcription factors that could account for much of the Treg signature [77]. Loss and gain of function experiments revealed that the Treg expression signature was robust; genetic deletion or retroviral overexpression of any of the co-factors individually had little effect, but overexpression of any one of a set of five factors (IRF4, GATA1, LEF1, SATB1 and Eos) together with FOXP3 lead to a strong synergistic establishment of the Treg signature. Consistent with the extent of the FOXP3 interactome, these factors were also found to interact with FOXP3 and act to enhance FOXP3 binding to already existing target sites to increase Treg gene induction [77].

The number of potential FOXP3 co-factors revealed by Rudra and colleagues [24], the ability of multiple factors to work with FOXP3 to generate the Treg expression signature as revealed by Fu *et al.* [77], and the extensive cross-regulation between FOXP3 and its co-factors revealed by both studies demonstrate that FOXP3 forms the core of a regulatory network containing multiple feedback loops and redundancy between factors. This may, therefore, allow robust induction of a Treg phenotype from different T cell states in varying immune environments [77]. Similar networks of factors have also been identified for Th17 cells [56, 78].

## Co-expression of lineage-specifying transcription factors

It had been considered that each helper T cell lineage had a corresponding master regulator factor that was necessary and sufficient for its differentiation, T-bet for Th1, GATA3 for Th2, FOXP3 for Treg and so on. The importance of the pioneer factors and the role of co-factors suggests that these ‘master’ regulators are not as all-powerful as perhaps thought and that they function within a complex regulatory network to define cell state. These master regulators should perhaps more correctly be referred to as lineage-specifying factors [79]. To confound matters further, it has emerged that expression of the lineage-specifying factors is not restricted to a single lineage but that they are frequently co-expressed with other lineage-specifying factors outside of their canonical subset (Table 1, [2, 79]). Although initially considered to constitute a transitional state during lineage commitment, co-expression of lineage-specifying factors also occurs in what appear to be stably committed subset populations [79]. A key challenge therefore is to understand how co-expression affects transcription factor function, and the consequences this has for cell phenotype and plasticity.

### Co-expression of FOXP3 with effector cell regulators—modulation of Treg properties

The effect of lineage-specifying factor co-expression is perhaps best understood in Tregs, in which the induction of effector lineage-specifying factors is necessary to target immunosuppressive activity appropriately. T-bet is upregulated in Tregs in response to IFNγ [25] or IL-27 [28]. These cells maintain their suppressive activity in both *scurfy* [25] and airway hyper-reactivity [26] mouse models with T-bet acting to induce the Th1 homing receptor CXCR3 [25]. Consistent with this, T-bet-positive Tregs accumulate at sites of Th1-mediated inflammation and T-bet is required for Treg homoeostasis and function during type 1 inflammation [25].

This role for lineage-specifying factors for Tregs to suppress inflammation associated with a specific effector subset is also demonstrated by the requirement for GATA3 for Treg accumulation at inflamed sites [23] and the increase in IL-4/5/13 producing Th2 cells and inflammatory disorders in mice in which *Gata3* is specifically deleted in FOXP3-positive cells [22, 24]. Similarly, FOXP3^+^RORγt^+^ cells express CD62L and traffic to the pancreas to suppress effector T cells in a type I autoimmune diabetes model [32], whereas FOXP3 + BCL6+ cells express the germinal-centre homing marker CXCR5 and limit the extent of the germinal centre reaction [33, 34]. Thus, FOXP3 appears to act dominantly, inducing a Treg expression program, while the co-expression of a lineage-specifying regulator, in response to the cytokine microenvironment, acts as a modulating agent to appropriately ‘polarize’ the suppressive activity.

What form does the interplay between FOXP3 and co-expressed lineage-specifying factors take? FOXP3 interacts with GATA3, with ChIP revealing GATA3 bound at sites at FOXP3 target genes [24]. Specific deletion of GATA3 in FOXP3-positive cells demonstrates that the two proteins can either act cooperatively or antagonistically, with GATA3 loss tending to lead to downregulation of their shared targets [24]. FOXP3 also interacts with RORγt, with FOXP3 acting dominantly, interfering with the ability of RORγt to activate its target genes [29, 30]. FOXP3 contains the sequence LQALL which matches the LxxLL interaction motif used by nuclear co-activators and co-repressors required for nuclear hormone receptor activity and therefore FOXP3 may act in a dominant negative fashion to prevent RORγt function [80]. The antagonism between FOXP3 and RORγt is also played out through physical competition for RUNX1, which is required for both Th17 and Treg differentiation [75, 81, 82]. Therefore, a common partner, such as a Runx protein, can provide the pivot through which transcription factor interplay is balanced (Figure 1D—Competition).

Although FOXP3 can act dominantly over the co-expressed effector regulator, changes in their relative levels may shift the balance towards an effector cell phenotype. FOXP3 repression of RORγt activity is released upon treatment with the Th17-inducing cytokines IL-6, IL-21 or IL-23 [30, 83]. Similarly, T-bet positive Tregs can gain effector function during *Toxoplasma gondii* infection [27]. Thus, although FOXP3 can act dominantly, the co-expression of effector regulators also provides a degree of plasticity between suppressor and effector function.

### Interplay between T-bet, GATA3 and RORγt

The co-expression between FOXP3 and the ‘master regulators’ of effector cell lineages fits within a model in which FOXP3 acts to impose suppressor function upon the defined effector subtypes. Co-expression of the classical lineage-specifying regulators T-bet, RORγt and GATA3 is more difficult to reconcile within a model in which each effector subtype is a distinct terminally differentiated lineage but may instead provide a mechanism for the functional plasticity that is apparent between T cell effector subtypes.

RORγt can be co-expressed with either GATA3 or T-bet and in both cases this appears to be associated with pathological outcomes. Th17 cells that co-express T-bet can be generated *in vitro* by culture in IL-6, IL-1β and IL-23 [44, 46] and their transfer into an allergic encephalomyelitis mouse model leads to more severe disease than conventional Th17 cells [44, 47, 84]. Similarly, Th17-producing Th2 cells were found to induce an influx of inflammatory leukocytes and to exacerbate asthma [48]. Microarray analysis shows that, similar to the role of T-bet in Treg homing, the co-expression of T-bet and RORγt creates a hybrid gene expression program, which includes expression of the Th1 homing marker CXCR3 [44, 47].

This blurring between the canonical effector lineages has even extended to the paradigmatic Th1 and Th2 subtypes. T-bet and GATA3 are co-expressed in *in vitro* differentiated primary human Th1 cells [39–41, 85] and in CCR5+ Th1 memory cells [42]. Furthermore, although *in vitro* polarized murine Th2 cells stably maintain a Th2 phenotype, transfer into mice subsequently infected with LCMV, or cultured *in vitro* with IL-12, IFNγ, IFNα + β and anti-IL-4, leads to co-expression of GATA3 and T-bet in these cells [21].

To understand the interplay between T-bet and GATA3 when they are co-expressed, we mapped their binding across the genome in primary human Th1 and Th2 cells using ChIP-Chip [40], and more recently ChIP-Seq [41]. We found that GATA3 exhibits a switch in its binding sites between Th2 and Th1 cells. GATA3 binds to a unique set of enhancer sites in Th2 cells and this is associated with Th2-polarized expression of the associated genes. In Th1 cells, GATA3 is distributed away from these positions and instead occupies a new set of sites at Th1 genes, which are also bound by T-bet (Figure 1E—Redistribution). Interestingly, although the Th2-specific sites contain a GATA motif, directly bound by GATA3, the Th1-specific sites do not, they only contain a T-box motif recognized by T-bet. Using a T cell line model in which T-bet and GATA3 could be expressed individually or together, we found that expression of T-bet is sufficient to induce GATA3 binding at Th1-specific sites, indicating it is directly responsible for the redistribution of GATA3 in Th1 cells [41]. Thus, T-bet would appear to act dominantly, sequestering GATA3 away from Th2 genes to prevent the activation of these genes in Th1 cells. This is consistent with the hypothesis that T-bet is primarily repressive and functions to block a default Th2 program [40, 86, 87].

How does T-bet cause a change in GATA3 binding? T-bet and GATA3 have been reported to directly interact [88] and the T-bet-GATA3 complex may only be able to bind to T-bet sites. Alternatively, T-bet may be able to influence GATA3 binding through RUNX3, which interacts with both T-bet [70] and GATA3 and is necessary for T-bet repression of *Il4* [89, 90]. T-bet antagonism of GATA3 at Th2 genes may be reflected by the reported ability of T-bet to block p300 binding at non-Th1 enhancer sites [55], which suggests that T-bet may act to decommission Th2 enhancers. As is the case for FOXP3 and its interaction partners, one could imagine that a change in the relative levels of T-bet or GATA3 could shift the balance and allow GATA3 to bind to Th2 sites. Thus, the maintenance of GATA3 expression in human Th1 cells, but in an alternative distribution, may allow expression of a Th1 phenotype while maintaining a degree of functional plasticity.

T-bet has a similar relationship with the Tfh-specifying factor BCL6. These two proteins are co-expressed during early Th1 development and in a subset of Tfh cells [35–38]. T-bet directly interacts with BCL6 and targets it to T-bet binding elements where the T-bet–BCL6 complex acts repressively (Figure 1F—Modulation). Interestingly, T-bet binds the BCL6 zinc finger DNA-binding domain, preventing BCL6 from contacting DNA at its canonical sites [37, 38]. Thus, T-bet may also interact with GATA3 through its zinc finger DNA-binding domain and thereby similarly occlude GATA3 DNA-binding activity. In cardiac cells, the T-box protein TBX5 interacts with GATA4 through its DNA binding domain [91], suggesting this form of interaction between T-box factors and co-expressed GATA family members is a common mechanism controlling mammalian developmental processes. In addition to its altered genomic distribution in Th1 cells, GATA3 also exhibits different patterns of binding in other CD4+ T cell lineages [92] and during earlier stages of T cell development [93], implying that other lineage-specific factors act to alter GATA3 binding to allow other changes in the T cell differentiation state. Analysis of motifs at these sites suggests that these other co-factors could be members of the Runx, Ets and AP-1 families [92]. Interestingly, GATA3 has different effects on gene activity and chromatin modification in different cells, suggesting that co-factors also act to modulate its activity [92]. Thus, the lineage-specific effects of GATA3 may primarily be a function of its interplay with other factors rather than a reflection of its own inherent activity.

## SUMMARY AND OUTLOOK

**Figure 2:**
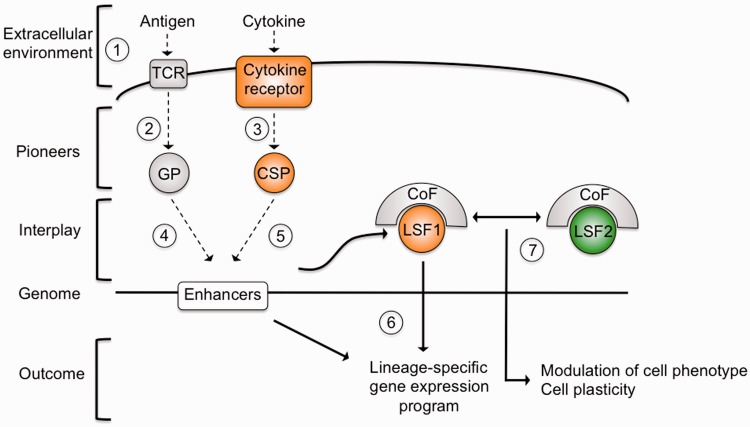
Summary of T cell differentiation control through transcription factor interplay. The extracellular environment is sensed by the cell through antigens and cytokines (1). TCR signalling leads to activation of general acting pioneer (GP) transcription factors, such as NFAT and AP-1 (2). In addition, the cytokine milieu causes activation of cytokine-specific pioneers (CSP), such as STATs (3). Together, these pioneers influence genome-wide enhancer competency (4) and the expression of a lineage-specifying factor (LSF1) (5). Interplay between the lineage-specifying transcription factor, co-factors and the pre-existing chromatin landscape results in a lineage-specific gene expression program (6). Transcription factors associated with other lineages (LSF2) may also be expressed (7), allowing modulation of the cell phenotype and cell plasticity.

Although limited to classifying cells by the expression of a handful of proteins, classical cellular immunological methods have been very useful in defining the archetypal helper T cell lineages and identifying the major signals and transcriptional regulators involved in their generation. However, the resultant model that helper T cells can differentiate into one of a number of distinct terminally-differentiated lineages has not been supported by more recent findings. Functional genomics methods are playing a key role in revealing the complex interplay that exists between helper T cell transcriptional regulators and the spectrum of cell phenotypes and plasticity that this creates (Figure 2).

Ligation of the TCR by antigen leads to activation of general pioneer factors such as NFAT and AP1 (Figure 2). These regulators act with STATs activated in specific patterns in response to cytokine signalling to prepare the activated T cell chromatin landscape. Different STATs lead to activation of different distal regulatory elements, providing a direct link between the cellular microenvironment and epigenetic regulation. Clearly, the microenvironment of a T cell *in vivo* will be much more complex than those used in *in vitro* differentiation models, leading to the activation of a correspondingly more complex transcriptional regulatory network and a more nuanced cell phenotype that reflects the balance of these input signals. The subsequent expression of lineage-specifying factors focuses cell fate, reinforcing a specific lineage choice while closing down potential alternative differentiation pathways. The lineage-specifying factors do not appear to have as fundamental effects on the epigenetic landscape as the pioneer factors, consistent with the requirement for the continued expression of these proteins to maintain cell state and the plasticity that is evident between lineages.

The signals received often lead to co-expression of more than one lineage-specifying factor. We are beginning to define the regulatory mechanisms that govern their interplay in these situations. There is clearly a regulatory ‘tug-of-war’ between co-expressed factors, often mediated by direct interaction that can lead to changes in transcription factor binding or activity. The relative levels between the factors would appear to be important in defining which factor ‘wins out’ but there also seems to be a regulatory hierarchy in which certain factors act dominantly over others. The outcome of these interactions is often a hybrid gene expression program, for example, allowing cells to take on features of more than one effector or to adopt a suppressor phenotype with a polarized homing activity (Figure 2). Changes in the cellular environment then allow for a change in the balance of power between co-expressed regulators. Thus, this mechanism not only provides a defined cell phenotype, but also allows the cell to respond to changing environmental conditions.

**Figure 3:**
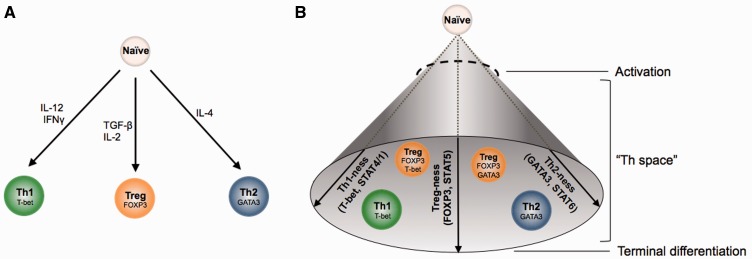
Changing consideration of different Th lineages to points within Th space. (**A**) The traditional view of T cell lineage specification downstream of ligation of the T cell receptor of naïve CD4+ T cells. Depending on the cytokine signals received, the cell can differentiate down one of several discrete pathways, leading to a set of distinct, non-overlapping T cell lineages, shown here for Th1, Th2 and Treg subtypes. Each lineage expresses a different master regulator transcription factor and produces a signature set of cytokines. (**B**) It may be more appropriate to consider naïve CD4+ T cells as having a wide range of possible fates, which can be classified according to the degree to which they are polarized along multiple axes, such as ‘Th1-ness’, ‘Th2-ness’ or ‘Treg-ness’, each of which is controlled by different network of factors. This creates a ‘Th-space’ in which the different cell phenotypes exist as relatively stable low energy points. Limited by the page, this space is shown here as a 3-dimensional cone, but the true number of potential dimensions along which a cell can polarize is not yet known. The degree to which a cell is polarized along each axis is a product of the balance of the signals received and the interplay between the resultant factors induced. Cells cross a differentiation boundary when activated (dashed circle), which they cannot re-cross, and migrate to a position within Th-space that dictates their phenotype. Cells maintain the potential to move within Th-space to adopt different phenotypes (plasticity) but may still be able to reach a stage of terminal differentiation at which their phenotype becomes fixed (represented by the plane at the end of the cone).

Considering these developments, it may not be appropriate to apply terms such as ‘Th1’, ‘Th2’ and ‘Treg’ to different cell lineages that can differentiate along distinct pathways from a naïve T cell (Figure 3A). It may instead be more helpful to consider ‘Th1’, ‘Th2’ and ‘Treg’ as properties or gene expression modules that cells can possess to varying extents (Figure 3B). For example, a FOXP3 + T-bet+ cell has both Treg and Th1 qualities. In this way, cells lie in different points within a multi-dimensional ‘Th space’ rather than at the ends of different linear pathways, with some points within this space being more stable (lower energy) than others (Figure 3B).

There is clearly much to do before we understand the mechanisms controlling transcription factor interplay, the differences in epigenetic modification and gene activity this causes and the resultant cell phenotypes produced. It will be necessary to combine reductionist approaches to define the mechanisms through which factors interact, the changes in gene activity this causes and then annotating these mechanisms onto system-wide views. These systems models could then be tested by disrupting individual regulator–regulator interactions in specific cell types.

A greater focus on the molecular mechanisms through which T cell transcription factors act may be of advantage. We often think of transcription factors as black boxes that bind DNA and activate or represses transcription but different factors achieve this in different ways and a greater knowledge of the specific epigenetic and transcriptional states affected by each factor will be of great use in understanding how their activities synergize or antagonize to generate a gene expression program. The study of factors such as T-bet, GATA3 and FOXP3 in other cell types will also aid the identification of the molecular events these factors control. By extending studies to innate lymphoid cells, can we define T-betness, beyond activation of *Ifng*? Is there something GATA3 does in both T cells and mammary luminal epithelium [94]? Does FOXP3 also interact with the same set of co-factors to modulate cell fate in the epithelium of the prostate, breast, lung and ovary [95]?

Technologies that will enable these advances include the ability to trace cell lineages *in vivo* and then characterize individual progeny cells using multi-colour-flow and single-cell RNA quantification [96]. This will be specifically important to define the potential cell phenotypes available from a given starting population, and to quantify their relative frequency and association with different microenvironments. Genome-wide sequential ChIP (ChIP-reChIP) will also identify the genomic sites at which different transcription factor complexes are positioned. Do FOXP3–IRF4 complexes bind at different sites to FOXP3–GATA3 complexes and do the different binding partners lead to different local histone modifications and associated changes in gene activity? The ability to perform ChIP-Seq on single cells is some way off but genetic modification to tag transcription factors in a cell-type-specific manner would allow transcription factor binding to be measured in a specific cell type when present within a mixed-cell population. The use of genomics methods to identify alterations to T cell states in human disease conditions and the identification of polymorphisms affecting transcription factor binding, will allow us to link pathology to changes in specific regulatory mechanisms. Such insights may also allow development of new strategies to alter the helper T cell differentiation state to relieve autoimmune or allergic conditions, to specify the phenotypes of adoptively transferred cells and enhance sub-optimal immune responses.

**Key Points**

- Regulation of helper T cell lineage specification is more complex and plastic than previously appreciated.
- T cell transcriptional factors tend to occupy common regulatory elements, with non-lineage specific factors and STATs acting as pioneers.
- Lineage-specifying regulators function with an extensive array of co-factors in complex regulatory networks.
- Lineage-specifying factors such as T-bet, GATA3 and FOXP3 are frequently co-expressed in different lineages and the balanced interplay between them dictates the resultant cell state.
- Different T cell lineages can be thought of as points within multi-dimensional ‘Th space’, defined by the degree to which they are polarized along different axes.

## FUNDING

This work was supported through an Oliver Bird Rheumatism Programme studentship, Lupus UK and the Wellcome Trust (091009).
